# Gallic acid’s protective mechanisms against acrylamide-induced pulmonary injury: in vivo and in silico insights into the Nrf-2/HO-1/NFκB pathway modulation

**DOI:** 10.1007/s00210-025-03996-1

**Published:** 2025-03-12

**Authors:** Merve Bolat, Samet Tekin, İsmail Bolat, Aslıhan Atasever, Burak Çinar, Yusuf Dağ, Emin Şengül, Serkan Yildirim, Mohamad Warda, Fikret Çelebi

**Affiliations:** 1 Department of Physiology, Atatürk University Faculty of Veterinary Medicine, Erzurum, Turkey; 2 Department of Pathology, Atatürk University Faculty of Veterinary Medicine, Erzurum, Turkey; 3 Veterinary Medicine, Çayırlı Vocational High School, Erzincan University, Erzincan, Turkey; 4 Department of Pharmacology, Atatürk University Faculty of Medicine, Erzurum, Turkey; 5 Department of Biochemistry, Faculty of Veterinary Medicine, Cairo University, Giza, Egypt

**Keywords:** Acrylamide, Gallic acid, Inflammation, In silico, Lung, Oxidative stress

## Abstract

Acrylamide (ACR) is a toxic compound formed during the heating of tobacco and starchy foods, contributing to increased reactive oxygen species (ROS) levels and significant health risks. This study evaluates the protective effects of gallic acid (GA), a natural polyphenol with potent antioxidant and anti-inflammatory properties, against ACR-induced lung injury. Fifty male rats were divided into five groups: Control, ACR, GA50 + ACR, GA100 + ACR, and GA100. Lung tissues were analyzed biochemically, histopathologically, immunohistochemically, and via immunofluorescence. GA exhibited dose-dependent protective effects by enhancing antioxidant defenses through Nrf-2 (43% increase) and HO-1 activation and reducing lipid peroxidation markers (MDA decreased by 38%). GA also suppressed pro-inflammatory mediators (TNF-α reduced by 35%) and restored anti-inflammatory levels by modulating the NF-κB pathway. Furthermore, GA reduced apoptosis (Caspase-3 activity decreased by 30%) and preserved lung tissue integrity by mitigating oxidative DNA damage (8-OHdG levels reduced by 29%) and pro-apoptotic signaling (Bax levels reduced by 34%). Computational analyses demonstrated GA's interaction with the KEAP1 protein, supporting its role in activating the KEAP1-Nrf2 pathway. These findings highlight GA's antioxidant, anti-inflammatory, and anti-apoptotic properties, suggesting its therapeutic potential for protecting against ACR-induced lung injury and paving the way for future research in lung health and toxicology.

## Introduction

Acrylamide (ACR) is a colorless, crystalline compound known for its high water solubility and widespread use in various industries such as textiles, cosmetics, mining, and construction (Su et al. 2024; Kahkeshani et al. 2015). It also serves as a key agent in water purification due to its capability to remove solid compounds (Peivasteh-Roudsari et al. 2024; Yousef and El-Demerdash 2006; Pennisi et al. 2013; Awad et al. 1998). Found not only in industrial settings but also in common dietary staples like bread, cereal, and French fries, ACR forms during high-temperature cooking processes involving carbohydrates (Koszucka et al. 2020; Yaylayan et al. 2003; Amrein et al. 2003), thereby extending exposure beyond occupational environments to everyday food consumption.

Despite its industrial utility, ACR poses significant health hazards as a probable human carcinogen, capable of inducing cancer upon exposure through diet, cigarette smoke, or occupational settings (Zamani et al. 2017). Metabolically, ACR undergoes partial conversion to glycidamide in the liver via Cytochrome P450 2E1 (CYP2E1), followed by conjugation with glutathione (GSH) to form mercapturic acid byproducts excreted in urine (Szczerbina et al. 2008; Uthra et al. 2017; Zamani et al. 2017). Glycidamide, more reactive than ACR, is implicated in multiple-organ failure by interacting with biomolecules like hemoglobin, enzymes, and DNA (Alturfan et al. 2012; Batoryna et al. 2019), inducing inflammation, apoptosis, and DNA damage in lung tissues (Abdel-Daim et al. 2020; Demir et al. 2022). ACR exposure also triggers lung inflammation, bronchiolar epithelial cell hypertrophy, and alveolar epithelial hyperplasia (Badavi et al. 2014), disrupting pulmonary redox homeostasis and elevating reactive oxygen species (ROS), leading to oxidative stress and subsequent acute lung injury (Tareke et al. 2008; Besaratinia and Pfeifer 2004; Catalgol et al. 2009; Sayed et al. 2022).

Given these health risks, various antioxidants, particularly plant-derived polyphenols, have been explored in animal models to mitigate ACR-induced lung injury (Choubey et al. 2015; Ghaznavi et al. 2018). Gallic acid (GA), also known as 3,4,5-trihydroxybenzoic acid, is a natural polyphenol abundant in fruits and tea leaves, celebrated for its potent antioxidant properties (Er et al. 2020; Badhani et al. 2015; Nikbakht et al. 2015; Kalantar et al. 2018). Beyond antioxidation, GA demonstrates anti-cancer, anti-inflammatory, anti-angiogenic, and antimicrobial activities, with potential benefits for respiratory ailments, allergies, lung fibrosis, COPD, and severe lung damage.

Recent studies have underscored GA's role in mitigating oxidative damage in biological systems (Hadidi et al. 2024; Badhani et al. 2015) and its protective effects against sepsis-induced lung injury in rats (Kardaş et al. 2023). Nevertheless, further exploration is needed to elucidate GA's potential in ameliorating ACR-induced pulmonary toxicity.

The purpose of this study is to determine the protective effect of GA against ACR-induced lung toxicity by biochemical, histopathological, immunohistochemical, and immunofluorescent methods. In addition, the possible interactions between GA and the Keap1 active site, antioxidant responses, and the effects of GA on the modulation of the Keap1-Nrf2 pathway against oxidative damage induced by ACR are also aimed to be investigated by in silico analysis.

## Methods and materials

### Chemicals

ACR (≥ 99%) (Cas No: 79–06-1) and GA (≥ 99%) (Cas No: 149–91-7) were supplied by Sigma-Aldrich Co. (St Louis, MO, USA). ELISA kits were sourced from BT Lab (MDA: [BT- LAB] (Cat.No: E0156Ra); SOD: [BT- LAB] (Cat.No: E0168Ra); GSH: [BT- LAB] (Cat.No: E0259Ra); GSH-Px: [BT- LAB] (Cat.No: E01172Ra); CAT: [BT- LAB] (Cat.No: E0869Ra); Nrf2: [BT- LAB] (Cat.No: E1083Ra); HO-1: [BT- LAB] (Cat.No: E0676Ra); TNF-α: [BT- LAB] (Cat.No: E0764Ra); IL-6: [BT- LAB] (Cat.No: E0135Ra);Il-1ß: [BT- LAB] (Cat.No: E0119Ra); IL-10: [BT- LAB] (Cat.No: E0108Ra); COX-2: [BT- LAB] (Cat.No: E0296Ra); iNOS: [BT- LAB] (Cat.No: E0740Ra); NF-κB: [BT- LAB] (Cat.No: E1817Ra);PGE2: [BT- LAB] (Cat.No: E0504Ra); Caspase-3: [BT- LAB] (Cat.No: E1648Ra); p38- MAPK: [BT- LAB] (Cat.No: E2473Ra); BAX antibody [Santa Cruz Biotecnology] (Cat No: sc-7480); 8-OHdG antibody [Santa Cruz Biotecnology] (Cat No: sc-66036); JNK antibody [Santa Cruz Biotecnology] (Cat No: sc-514539); FITC secondary antibody [Abcam] (Cat No: ab6785); Texas Red secondary antibody [Abcam] (Cat No: ab6719); DAPI [Thermo] (Cat No: D1306).

### Animals

Sprague Dawley rats used in this study were sourced from the Atatürk University Medical Experimental Research and Application Center (ATADEM). This study protocol was approved by Atatürk University Animal Experiments Local Ethics Committee (Approval No: 2023/ 88). A total of 50 male rats with an average weight ranging from 200 to 250 g were included. Rats were housed in an environment with a temperature set to 25°C, humidity maintained at 60% ± 10%, and a 12-h light/dark cycle. They had ad libitum access to both water and pellet feed. A power analysis program was used to determine the number of animals in the group. It was determined that at least 10 rats were needed in each group and at least 50 rats in total, with a 0.05 error (Type I, α) for 95% power (Type II error, β). Data from a previous study were used for this analysis (Erdemli et al. 2022).

### Experimental design

Prior to the start of the experiment, all rats were weighed and randomly assigned to one of five groups:

Group I (Control): Intragastric (IG) administration of 1 ml ddH_2_O for 11 days; Group II (ACR): IG administration of ACR at a dose of 50 mg/kg (He et al. 2017) for 11 days; Group III (ACR + GA50): Administration of GA at a dose of 50 mg/kg (Eslamifar et al. 2021; Dehghani et al. 2020; Alazragi 2020) IG, followed by administration of ACR at the same dose for 11 days; Group IV (ACR + GA100): Administration of GA at a dose of 100 mg/kg (Eslamifar et al. 2021; Dehghani et al. 2020; Alazragi 2020) IG, followed by administration of ACR at the same dose for 11 days; Group V (GA100): IG administration of GA at a dose of 100 mg/kg/day for 11 days.

For Groups III and IV, GA was administered 1 h before ACR each day. On the 12th day, the rats were weighed again and then euthanized under sevoflurane anesthesia. Lung tissues were harvested, weighed, rinsed with physiological saline, and stored at −80°C for subsequent analyses.

### Homogenization of lung tissue

Equal amounts of lung tissue samples were weighed and transferred to screw-capped tubes. The tissues were homogenized using a Magna Lyser homogenizer by adding 1500 µl of phosphate-buffered saline (PBS) solution. The homogenization was performed at 5000 rpm for approximately 10 min. Following homogenization, the samples were centrifuged at 5000 rpm for 10 min. The resulting supernatants were carefully transferred to clean Eppendorf tubes for further analysis.

### Biochemical analyses

#### Oxidative parameters and antioxidant enzymes analysis

Oxidative parameters and antioxidant enzyme activities were measured using an ELISA plate reader (Bio-Tek, Winooski, VT, USA) set to read absorbance at 450 nm. Measurements included malondialdehyde (MDA), glutathione peroxidase (GSH-Px), superoxide dismutase (SOD), glutathione (GSH), nuclear factor erythroid 2-related factor 2 (Nrf2), catalase (CAT), and heme oxygenase-1 (HO-1), following the protocols provided by the respective ELISA kits on the previouisly recovered supernatants.

#### Inflammation markers analysis

Analysis of inflammation markers including interleukin-1 beta (IL-1β), interleukin-6 (IL-6), tumor necrosis factor alpha (TNF-α), interleukin-10 (IL-10), nuclear factor kappa-B (NF-κB), cyclooxygenase-2 (COX-2), inducible nitric oxide synthase (iNOS), and prostaglandin E2 (PGE2) in the supernatants was conducted following the protocols provided by the respective ELISA kits.

### Histopathological examination

Following the conclusion of the experiment, tissue samples were immersed in a 10% neutral buffered formalin solution for 48 h and underwent standard tissue processing procedures before being embedded in paraffin blocks. Sections of 4 µm thickness were cut from each block, stained with hematoxylin–eosin (HE), and examined under a light microscope (Olympus BX 51, Camera DP72, Japan) for detailed histopathological analysis. Histopathologically, degeneration and necrosis in bronchial bronchioles and alveoli epithelial cells as well as inflammation in the tissue were evaluated in lung tissues. Evaluation of tissue sections was based on histopathological criteria, an area was evaluated as none cells (-), 1–5 cells ( +), 6–15 cells (+ +) and 16 < cells (+ + +) (Renne et al. 2009; Karabulut Uzunçakmak et al. 2023).

### Immunohistochemical examination

Tissue sections affixed to poly-L-lysine-coated slides for immunoperoxidase analysis were deparaffinized and dehydrated. Endogenous peroxidase activity was quenched by treatment with 3% H_2_O_2_ for 10 min. Antigen retrieval was performed by boiling the sections in 1% antigen retrieval solution (citrate buffer, pH 6.1), followed by cooling to room temperature. To minimize nonspecific staining, sections were incubated with protein block for 5 min. Primary antibody against BAX (Cat No: sc-7480, Dilution: 1/100, USA) was applied and incubated as per the manufacturer's guidelines. Chromogen 3,3'-Diaminobenzidine (DAB) was employed for chromogenic visualization. Stained sections were examined under a light microscope (Zeiss AXIO, Germany) (Bolat et al. 2024; Cicek et al. 2022).

### Double immunofluorescence examination

Tissue sections mounted on poly-L-lysine-coated slides for immunofluoresence analysis underwent deparaffinization and dehydration. Antigen retrieval was performed by boiling in 1% antigen retrieval solution (citrate buffer, pH 6.1) and allowed to cool to room temperature. To minimize nonspecific staining, sections were treated with protein block for 5 min. Primary antibody against 8-OHdG (Cat No: sc-66036, Dilution: 1/100, USA) was applied and incubated according to the manufacturer's instructions. A secondary immunofluorescence antibody (FITC, Cat No: ab6785, Dilution: 1/1000) was used for 45 min in the dark. Subsequently, primary antibody against JNK (Cat No: sc-514539, Dilution: 1/100, USA) was applied and incubated as per instructions. Another secondary immunofluorescence antibody (Texas Red, Cat No: ab6719, Dilution: 1/1000, UK) was used for 45 min in the dark. Finally, DAPI with mounting medium (Cat No: D1306, Dilution: 1/200, UK) was applied for 5 min in the dark, followed by coverslipping. Stained sections were examined under a fluorescence microscope (Sulukan et al. 2023; Bolat et al. 2025).

### In Silico analysis of GA via KEAP1/NRF2/ARE

This study utilized Schrödinger Maestro 2023/1 version to investigate the effects of GA on the KEAP1/NRF2/ARE pathway. The following detailed procedures were conducted:

#### Molecular docking

The interactions of GA with KEAP1, NRF2, and ARE components were meticulously modeled using Schrödinger Maestro 2023/1 version. The obtained binding results were carefully analyzed to determine interaction energies and potential binding positions (Hasanat et al. 2017).

#### 2D, 3D, and surface imaging

Molecular docking results were visualized using Maestro's 2D drawing tools, and interaction regions were meticulously identified. 3D examination of molecular structures was conducted using Maestro's advanced visualization tools, enhancing our understanding of the spatial patterns of binding interactions and providing improved visual representation (Chaudhari and Bari 2016).

#### MM-GBSA calculation

Based on the docking results, MM-GBSA calculations were performed to evaluate the thermodynamic stability of interactions of GA with KEAP1, NRF2, and ARE components (Fatima et al. 2023).

#### Pharmacophore mapping

The pharmacophores of GA on the KEAP1/NRF2/ARE pathway were identified using Maestro's pharmacophore mapping tools (Kumar et al. 2019).

#### Molecular dynamics simulation

To validate docking and MM-GBSA outcomes, Schrödinger Maestro's Molecular Dynamics Simulation module conducted extensive simulations. The dynamics of the KEAP1/NRF2/ARE complex with GA were scrutinized in this in silico analysis, facilitated by the robust capabilities of Schrödinger Maestro 2023/1 version (Cai et al. 2021).

### Statistical analysis

Statistical analyses were conducted using SPSS 20.00 software for studies involving more than two independent groups. One-way ANOVA was employed to assess quantitative values, followed by Tukey's test for post hoc comparisons, with results reported as mean ± standard deviation and significance set at *P* < 0.05.

For histopathological evaluations, SPSS 13.0 was utilized with significance defined as p < 0.05. Duncan's test facilitated intergroup comparisons, while the Kruskal–Wallis test detected group interactions and the Mann–Whitney U test determined differences between groups.

Quantification of immunohistochemical and immunofluoresence staining intensity utilized ZEISS Zen Imaging Software, evaluating five random areas per image. Data were expressed as mean ± standard deviation (SD) for percentage area. Statistical analyses included one-way ANOVA followed by Tukey's test using GraphPad Prism, comparing immunoreactive cell counts and stained areas with controls.

## Results

### Effect of ACR and GA applications on starting, ending weights, and lung weights of rats

There were no significant differences in body weights among the experimental groups on the first day of the study. Upon completion of the experiment, a reduction in body weight was observed across all groups subjected to ACR intoxication and subsequent treatment. The animals in the control group specifically exhibited significantly greater body weights compared to those in the ACR-treated groups. While lung weights were relatively lower in the ACR, GA50 + ACR, and GA100 + ACR groups compared to the control group, this disparity did not achieve statistical significance. In addition, when the average body weights of the rats were examined, no significant difference was found between the groups. Again, when the average body weight was compared to the weights of the lung tissues, no significant difference was found between the groups (*p* > 0.05) (Table 1).

**Table 1:** Effects of ACR and GA on the starting, ending, and lung weights of rats (*n* = 10)

Parameters	Groups				
	Control	ACR	GA50 + ACR	GA100 + ACR	GA100
Initial body weights (g)	241.53 ± 25.56^a^	235.41 ± 16.32^a^	232.86 ± 16.18^a^	226.86 ± 14.17^a^	223.29 ± 12.03^a^
Final body weights (g)	264.57 ± 15.24^a^	206.57 ± 24.96^b^	218.86 ± 12.32^b^	232.86 ± 18.32^ab^	236.14 ± 13.71^ab^
Average Body Weight	253.05 ± 20.4^a^	220.99 ± 20.64^a^	225.86 ± 14.25^a^	229.86 ± 16.24^a^	234.21 ± 12.87^a^
Lung weights (mg)	1.70 ± 0.0.14^a^	1.24 ± 0.11^b^	1.51 ± 0.03^ac^	1.53 ± 0.14^ac^	1.57 ± 0.10^ac^
Lung Weight / Average Body Weight	0.006 ± 0.006^a^	0.005 ± 0.005^a^	0.006 ± 0.002^a^	0.006 ± 0.008^a^	0.006 ± 0.007^a^

Letters other than 'a' on the same line indicate a statistically significant difference at *p* < 0.05. ± SEM

### Oxidative stress parameters in lung tissue

The activities of SOD, GPx, and CAT were significantly reduced in the ACR group compared to the control, GA100 + ACR, and GA100 groups. Treatment with GA resulted in a dose-dependent increase in these enzyme activities, with a particularly notable effect observed in the high-dose GA group. Evaluation of MDA levels revealed a significant increase in the ACR group compared to other groups; however, GA treatment led to a dose-dependent reduction in MDA levels. Significant decreases in GSH levels were also observed in the ACR group compared to other groups. Nrf-2 and HO-1 activities in lung tissue were significantly lower in the ACR group compared to the control and high-dose GA groups. Treatment with low-dose GA resulted in increased Nrf-2 and HO-1 activities compared to the ACR group, with a more pronounced effect observed at higher GA doses. Notably, the 100 mg/kg dose of GA restored values similar to those of the control group (Table 2).

**Table 2:** Effects of GA against ACR-induced oxidative stress in rats (*n* = 10)

Parameters	Control	ACR	GA50 + ACR	GA100 + ACR	GA100
SOD (ng/mL)	4.78 ± 0.67^a^	2.78 ± 0.6^b^	3.9 ± 0.59^a^	4.91 ± 0.67^a^	4.83 ± 0.51^a^
MDA (ng/mL)	1.95 ± 0,17^a^	3.05 ± 0,23^b^	2.34 ± 0.15^b^	1.99 ± 0.43^a^	1.93 ± 0.21^a^
GPx (ng/mL)	1326 ± 298.81^a^	643.01 ± 320.15^b^	876.11 ± 211.60^a^	1278.94 ± 218.71^a^	1410.44 ± 170.66^a^
GSH (ng/mL)	6.87 ± 1.63^a^	2.75 ± 1.09^b^	4.12 ± 1. 82^a^	6.61 ± 1.54^a^	6.51 ± 1.08^a^
CAT (ng/mL)	61.61 ± 15.76^a^	34.52 ± 9.23^b^	47.86 ± 11.68^a^	56.02 ± 16.13^c^	58.76 ± 16.30^a^
Nrf-2 (ng/mL)	1492.43 ± 452.05^a^	626.84 ± 112.78^b^	1005.51 ± 323.56^a^	1481.55 ± 324.05^a^	1662.20 ± 267.61^a^
HO-1 (ng/mL)	8.65 ± 1.31^a^	4.78 ± 1.07^b^	6.50 ± 1.70^a^	8.24 ± 0.63^a^	8.61 ± 1.69^a^

Letters other than 'a' on the same line indicate a statistically significant difference at *p* < 0.05. ± SEM

### Parameters related to inflammation in lung tissue

**Fig. 1:**
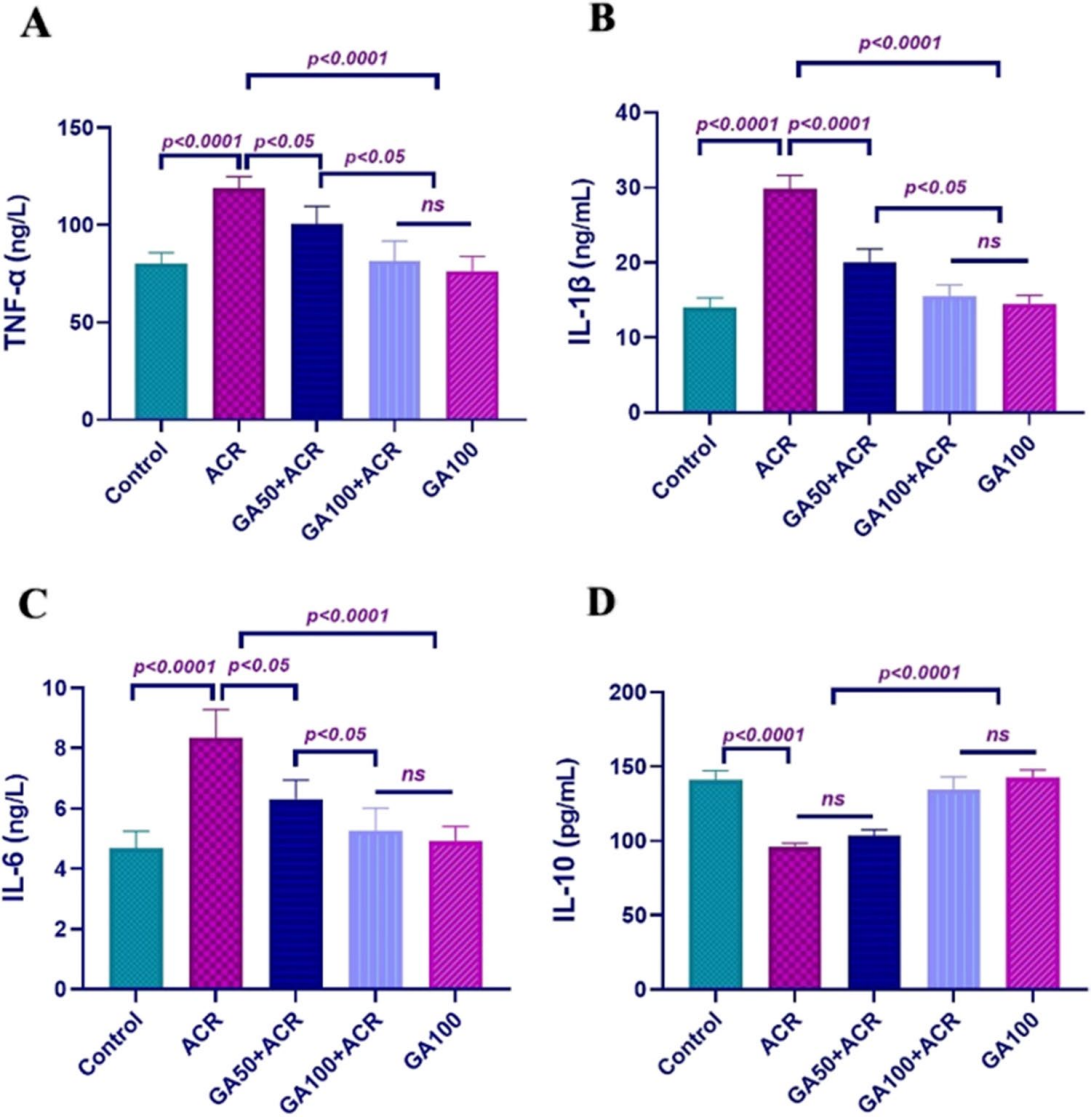
Illustrates the levels of inflammatory mediators TNF-α (**A**), IL-1β (**B**), IL-6 (**C**), and IL-10 (**D**) in lung tissues (*n* = 10). Results are expressed as mean ± SD

Based on the assessments depicted in Fig. 1, the ACR-exposed group exhibited substantial increases in proinflammatory mediators TNF-α, IL-1β, and IL-6 compared to both control and high-dose GA-treated groups. Treatment with GA demonstrated a dose-dependent reduction in these mediators, with the highest efficacy observed in the high-dose GA group. Notably, the 100 mg/kg GA dose normalized these values similar to the control group. Evaluation of IL-10 levels revealed a significant decrease in the ACR group, which showed a dose-dependent reversal with GA treatment.

**Fig. 2:**
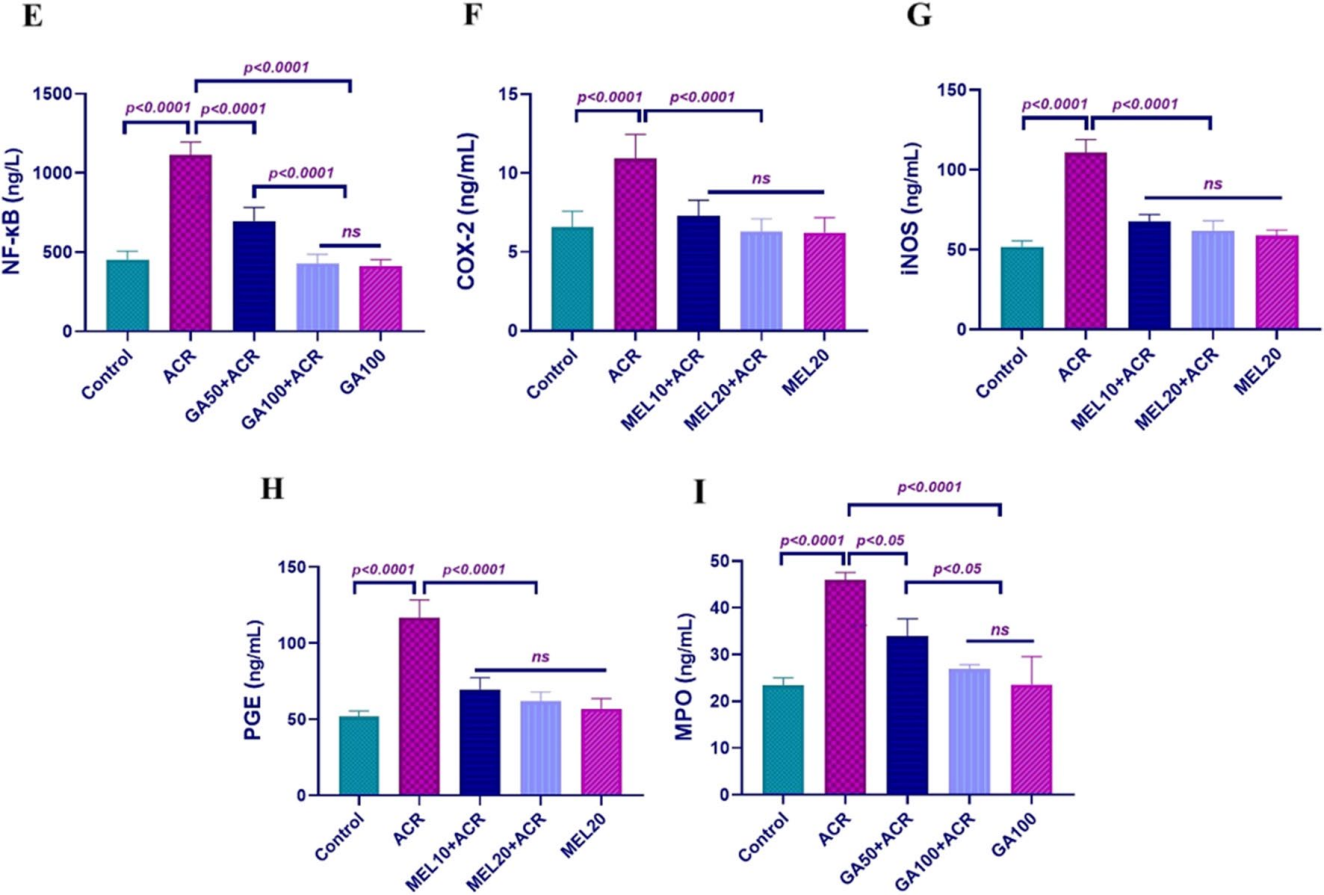
Illustrates NF-κB (**E**), COX-1 (**F**), iNOS (**G**), PGE2 (**H**), and MPO (**I**) levels in lung tissues (*n* = 10). Results are expressed as mean ± SD

In Fig. 2, significant elevations in NF-κB, iNOS, and MPO levels were observed in the ACR-exposed group compared to both the control and high-dose GA groups. Treatment with increasing doses of GA resulted in a dose-dependent reduction in NF-κB, iNOS, and MPO activities, with the highest effect seen in the high-dose GA group. The 100 mg/kg GA dose markedly normalized these activities to levels similar to the control group. Evaluation of COX-1 and PGE2 levels revealed a significant decrease in the ACR group compared to other groups, which was prevented by GA treatment in a dose-dependent manner.

### Apoptosis parameters in lung tissue

**Fig. 3:**
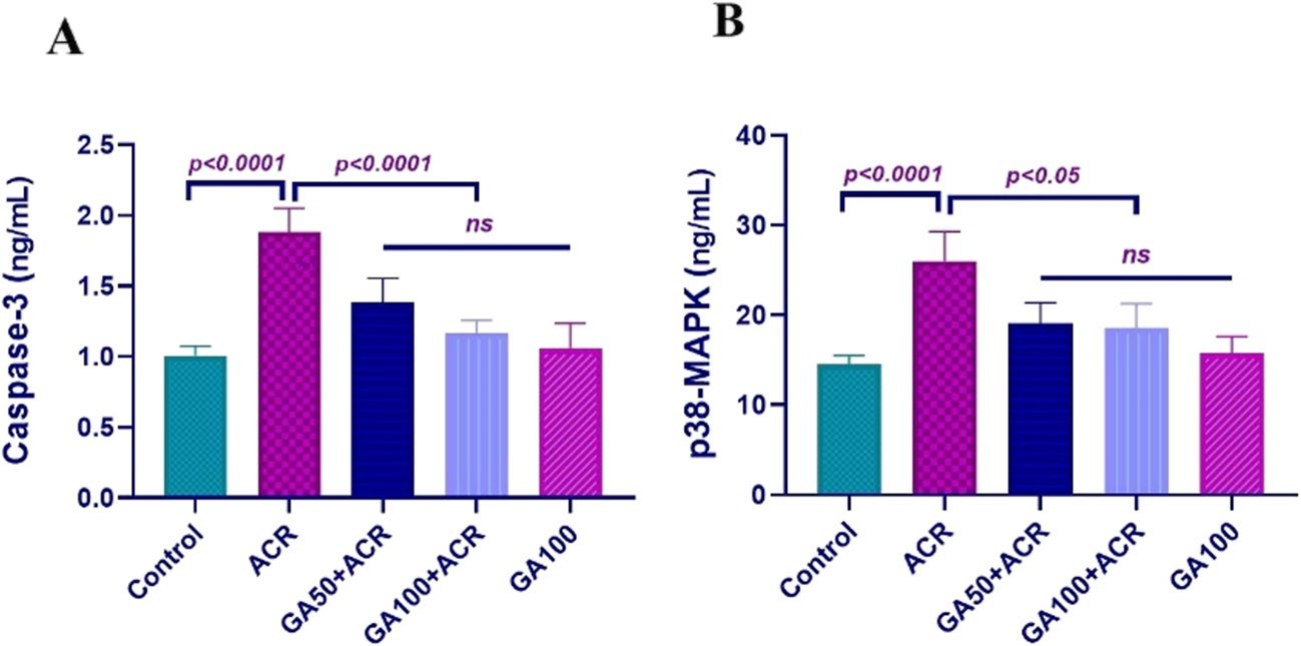
Represents Caspase-3 (**A**) and p38-MAPK (**B**) levels in lung tissues (*n* = 10). Results are expressed as mean ± SD

Figure 3 illustrates the outcomes of the evaluations conducted on Caspase-3 and p38-MAPK enzyme activities. In the ACR group, significant increases were observed compared to both the control and high-dose GA groups. Treatment with GA resulted in a dose-dependent reduction of these mediators, with the most pronounced decrease observed in the high-dose GA group. Administration of GA at 100 mg/kg restored these enzyme activities to levels comparable to those observed in the control group. These findings underscore GA's potential to attenuate ACR-induced changes in apoptotic and MAPK signaling pathways, highlighting its therapeutic promise in mitigating cellular stress and apoptosis in lung tissues.

### Histopathological findings

**Fig. 4:**
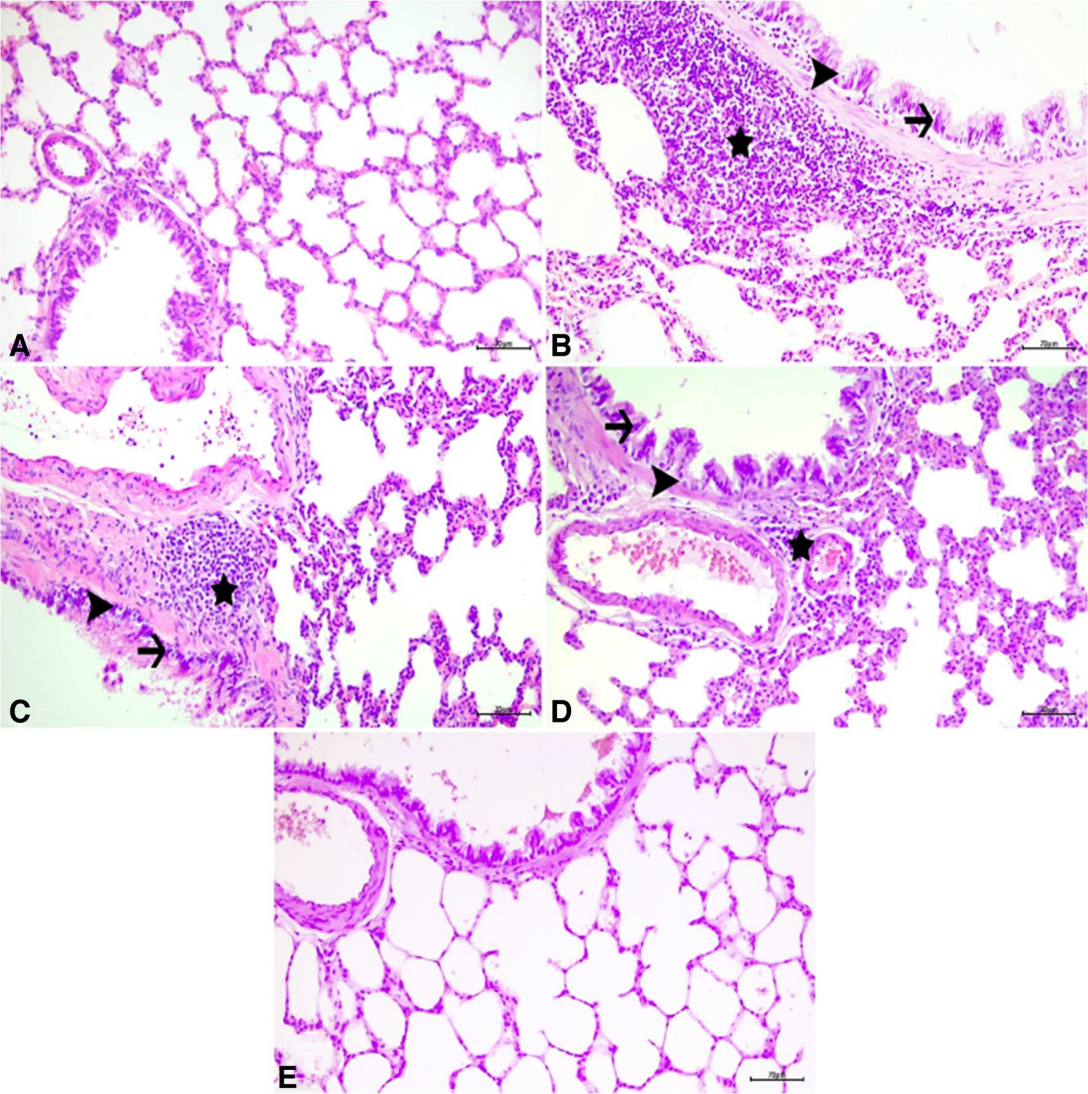
Lung tissue sections stained with H&E: **a** Control group showing normal histological structure. **b** ACR group exhibiting degeneration (arrowheads) and necrosis (arrows) in bronchial epithelial cells, with mononuclear cell infiltrates (asterisk). **c** ACR + GA50 group with similar pathological changes but less severe compared to ACR group. **d** ACR + GA100 group showing mild degeneration and necrosis in bronchial epithelial cells, with minimal mononuclear cell infiltration. **e** GA100 group showing normal histological structure similar to the control group. Scale bar = 70 µm

Figure 4 presents the histopathological examination results of lung tissues from different experimental groups. Control and GA100 groups showed normal histological structures (Fig. 4a and e). In contrast, the ACR group exhibited severe degeneration and necrosis in bronchial, bronchiolar, and alveolar epithelia, along with pronounced mononuclear cell infiltration around bronchi and interalveolar regions, leading to significant interalveolar septum thickening (Fig. 4b). Similar observations of degeneration, necrosis, and mononuclear cell infiltration were noted in the ACR + GA50 (Fig. 4c) and ACR + GA100 groups (Fig. 4d), although to a lesser extent compared to the ACR group. Since toxicity did not occur directly through inhalation in histopathological analyses, non-proliferative lesions were observed in the results. These findings were statistically significant compared to the ACR group. Detailed histopathological scoring is provided in Table 3, and corresponding statistical analyses are depicted in Fig. 4.

**Table 3:** Histopathological findings and scoring of lung tissues (*n* = 10)

Parameters	Groups				
	Control	ACR	GA50 + ACR	GA100 + ACR	GA100
Epithelial cell degeneration	-	+ + +	+ + +	+ +	-
Necrosis in epithelial cells	-	+ + +	+ +	+	-
Mononuclear cell infiltrations	-	+ + +	+ + +	+	-

none (-), mild ( +), moderate (+ +) and severe (+ + +)

### Immunohistochemical and immunofluoresence findings

**Fig. 5:**
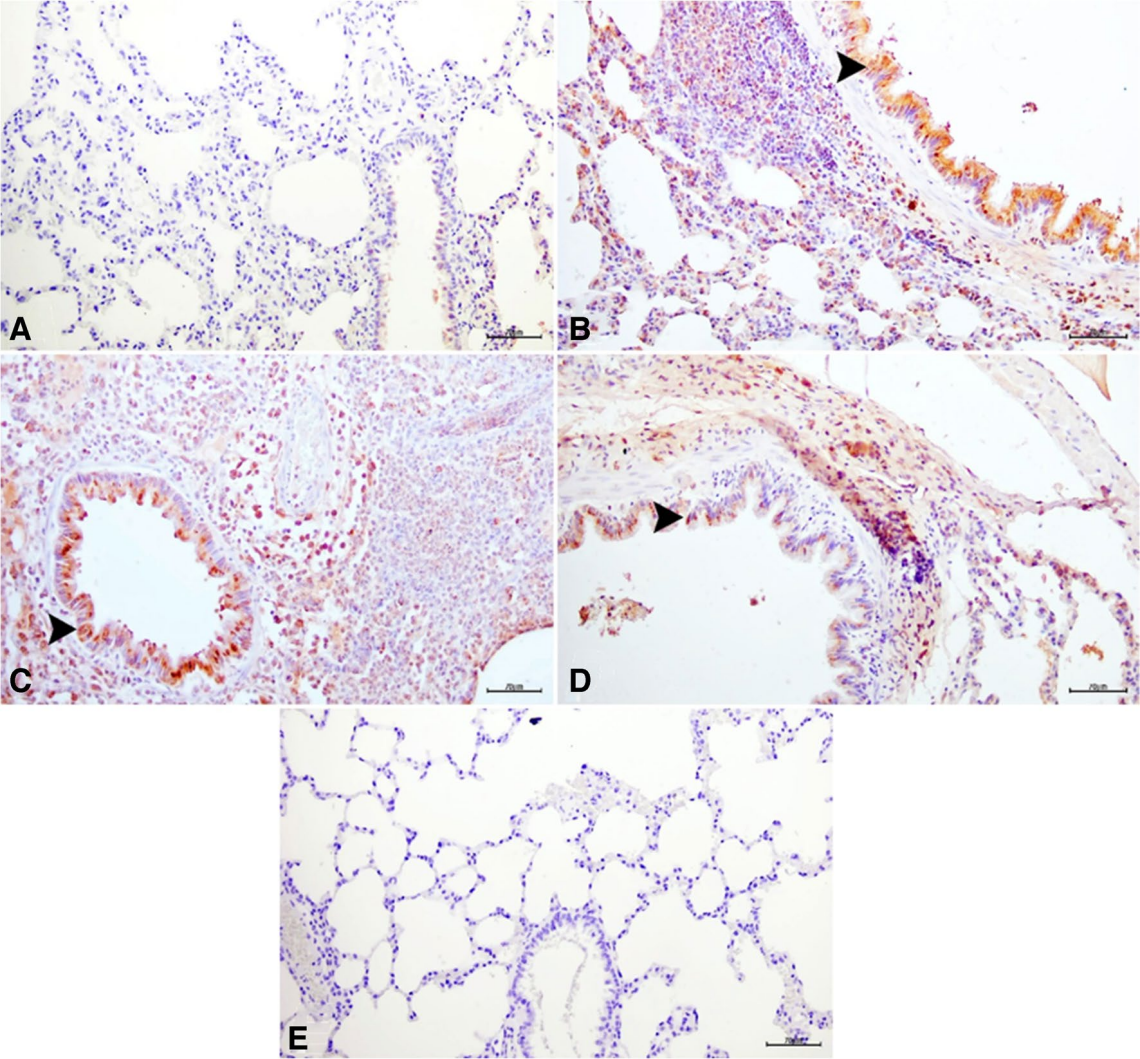
Lung tissue samples from different experimental groups: Control (**a**), ACR (**b**), ACR + GA50 (**c**), ACR + GA100 (**d**), and GA100 (**e**). The images depict BAX expressions (arrowheads) in bronchial, bronchiolar, and alveolar epithelial cells, visualized through immunohistochemical staining. Scale bar, 70 µm

**Fig. 6:**
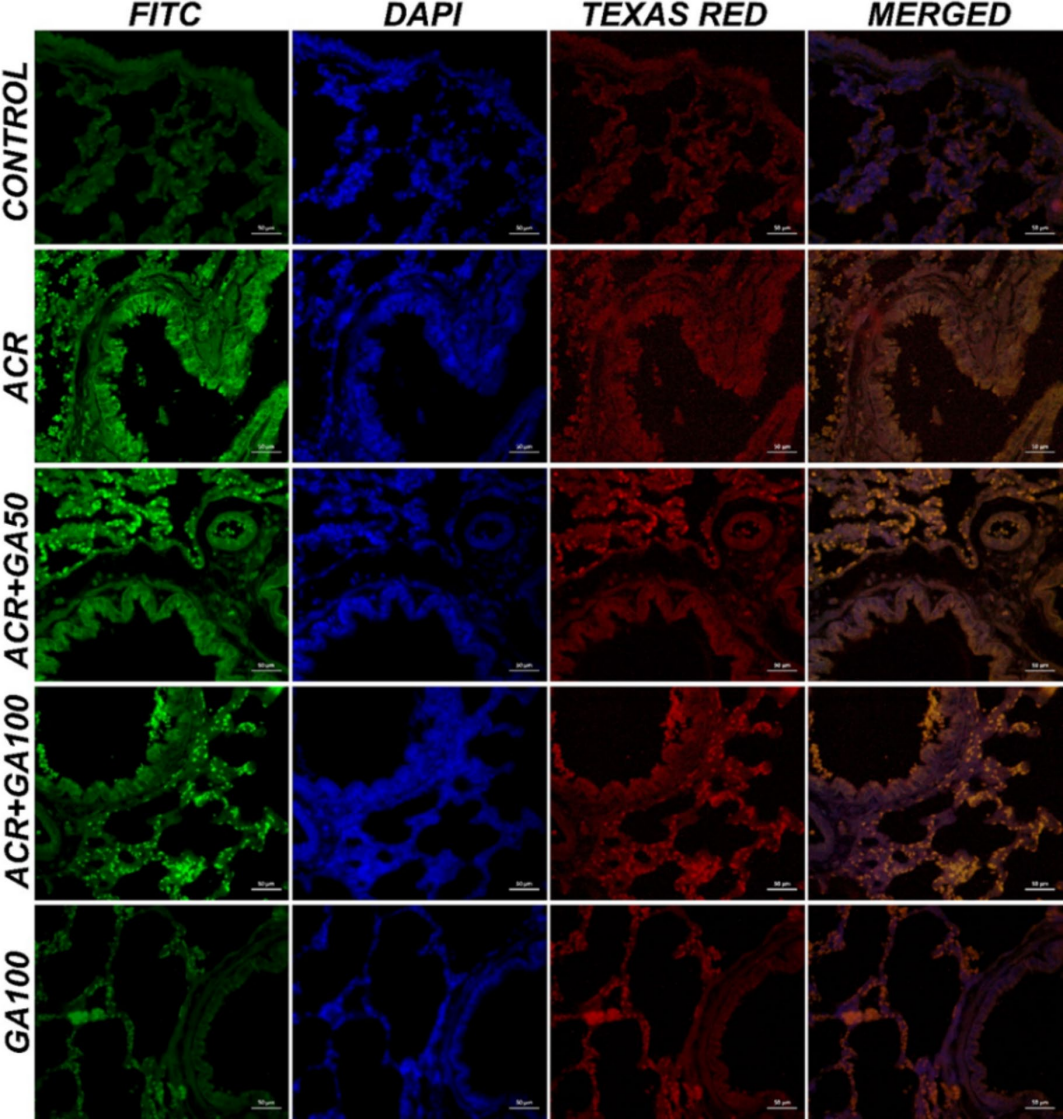
The immunofluorescent (IF) staining of 8-OHdG expressions (FITC) and JNK expressions (Texas Red) in lung tissue, specifically in bronchial, bronchiolar, and alveolar epithelial cells. Scale bar, 50 µm

**Fig. 7:**
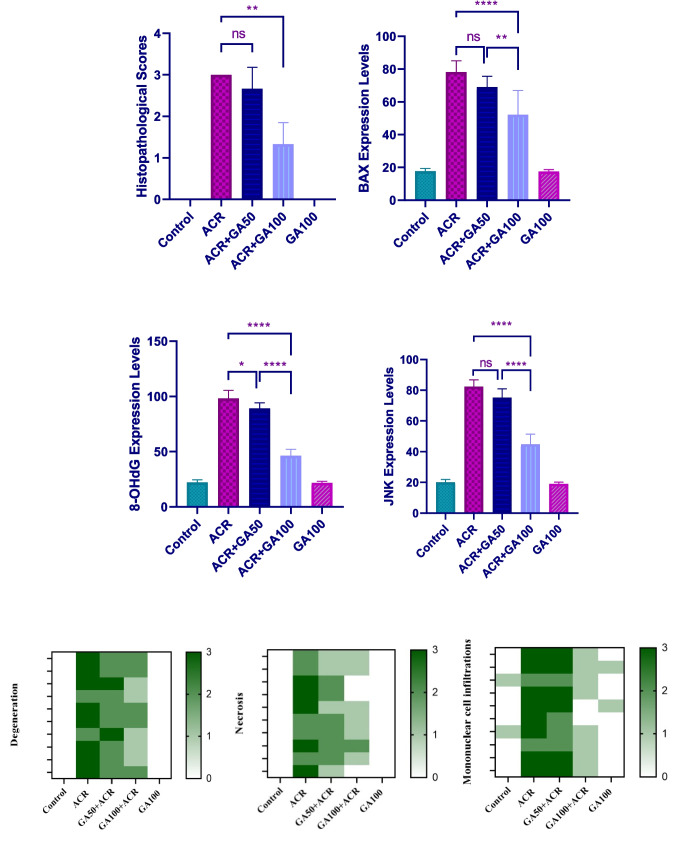
Histopathological findings in lung tissues (ns: no significant difference (*p* = 0.4545), **: *p* < 0.05 (*p* = 0.0022)), immunohistochemical (BAX, ns: no significant difference (*p* = 0.2944), **: *p* < 0.05 (*p* = 0.0084), ****: *p* < 0.05 (*p* = < 0.0001)), and immunofluorescence findings (8-OHdG *: *p* < 0.05 (*p* = 0.0302), ****: *p* < 0.05 (*p* = < 0.0001)) and (JNK ns: no significant difference (*p* = 0.0715), ****: *p* < 0.05 (*p* < 0.0001)). Graphs showing the specific severity of histopathological findings according to animals (Degeneration, necrosis and mononuclear cell infiltration) (*n* = 10)

In the immunohistochemical and immunofluorescence analyses of lung tissues, as depicted in Figs. 5a, b, c, e, and 6, BAX, 8-OHdG, and JNK expressions were evaluated across different experimental groups. The control and GA100 groups showed negative expressions of BAX, 8-OHdG, and JNK (Figs. 5a, e, 6a). In contrast, the ACR group exhibited severe intracytoplasmic BAX expression in bronchial, bronchiolar, and alveolar epithelial cells (Fig. 5b), along with significant level of expression of 8-OHdG and JNK (Fig. 6). The ACR + GA50 group demonstrated moderate intracytoplasmic BAX expression (Fig. 5c) and moderate levels of 8-OHdG and JNK (Fig. 6). Similarly, the ACR + GA100 group showed mild intracytoplasmic BAX expression (Fig. 5) and comparable level of expression of 8-OHdG and JNK (Fig. 6) to the ACR + GA50 group. These findings are presented alongside statistical analyses in Fig. 7.

### Molecular docking results

In the molecular docking study using the Schrödinger Maestro program, GA successfully docked into the active site of the KEAP1 protein. During the docking process, it was observed that the ligand formed hydrogen bonds with the ARG 415 amino acid of KEAP1. Additionally, the ligand established two hydrogen bonds each with SER 363, ASN 414, and SER 602 amino acids, resulting in a total of five hydrogen bonds with GA. These interactions highlight stable hydrogen bond formations between specific amino acids of KEAP1 and GA. Specifically, ARG 415, SER 363, ASN 414, and SER 602 were identified as key binding sites for GA within the active site. These findings emphasize GA's potential to effectively interact with the KEAP1 protein, suggesting its antioxidant properties could contribute to the KEAP1-NRF2 antioxidant pathway.

### Pharmacophore mapping analysis

**Fig. 8:**
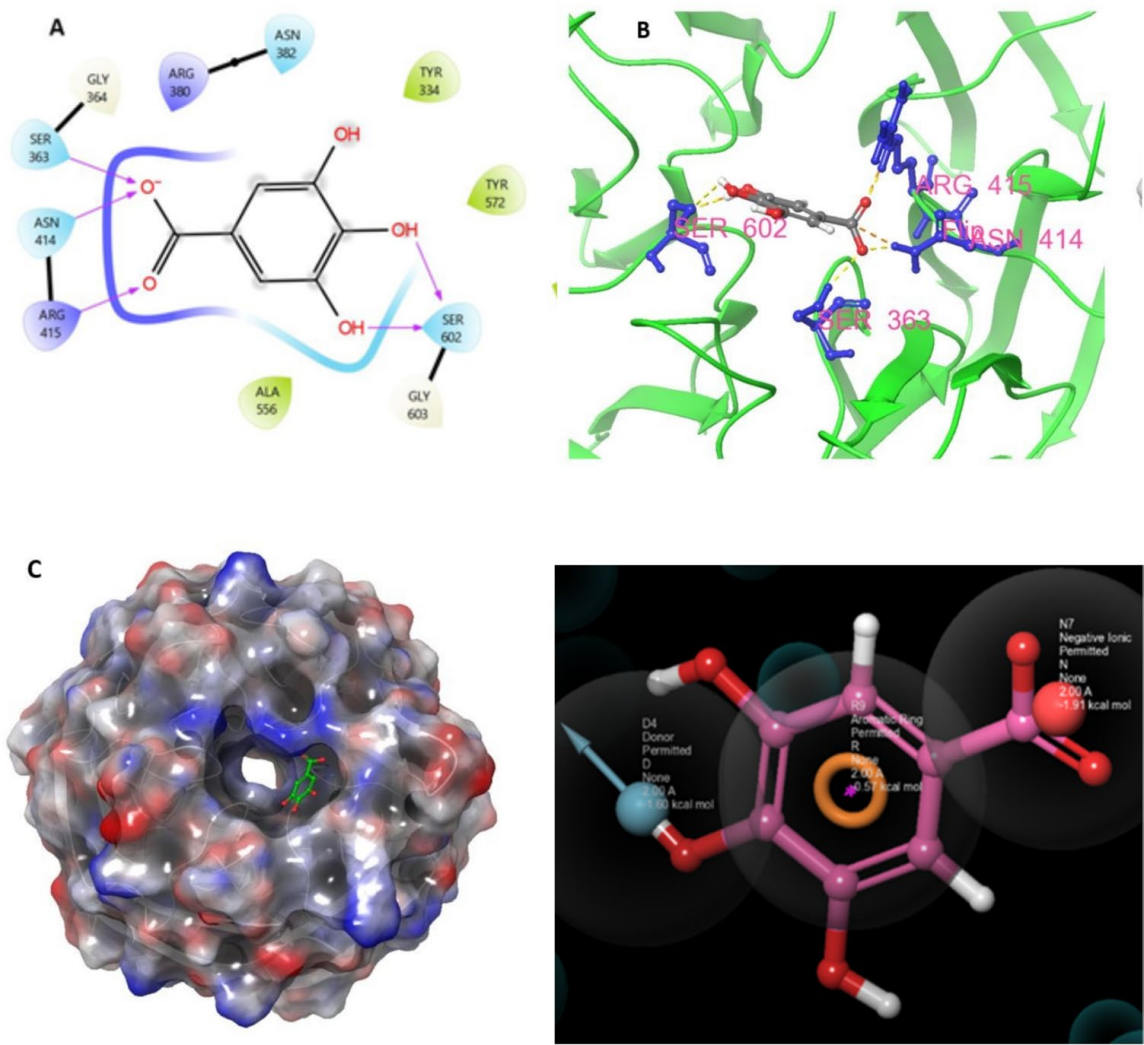
**A** 2D Representation of Molecular Docking, **B** 3D Representation of Molecular Docking, **C** Surface View of Molecular Docking, **D** Pharmacophore Mapping (Images generated using Schrödinger Maestro 2023/1 Licensed Version). 3.9. MM-GBSA Analysis Results (GA)

In pharmacophore mapping, specific molecular features are systematically identified to elucidate how a ligand interacts with a biological target. In this study, critical interactions were observed: the donor feature at position D4 exhibited an energy of −1.60 kcal/mol, the aromatic ring at position R9 had an energy of −0.57 kcal/mol, and the negative ionic structure at position N7 displayed an energy of −1.91 kcal/mol. These findings elucidate key molecular interactions essential for understanding the ligand's binding affinity and potential interactions with the biological target. Such insights are pivotal for guiding the development of more targeted and potent compounds during the drug design process (Fig. 8).

Bond Energy: 1.65 kcal/mol, Angle Energy: 2.10 kcal/mol, Torsional Energy: 0.16 kcal/mol, Electric Energy (EL): −21.16 kcal/mol, Lennard–Jones (LJ) Energy: 11.72 kcal/mol, Solvatation Energy (SGB): −8.66 kcal/mol, Nonpolar Energy: 0.00 kcal/mol, Lipophilic Energy (LIPO): 0.00 kcal/mol, Hydrogen Bond (Hbond) Energy: 0.00 kcal/mol, Pi-Pack Energy: 0.00 kcal/ mol, Self-Contact Energy: 0.00 kcal/mol, Total Energy (TOT): −14.18 kcal/mol.

These results indicate that GA binds stably, specifically stabilized by electrostatic interactions and Lennard–Jones interactions. The negative total energy value suggests that this component contributes to the stability of the complex (Table 4).

### GA ADME/ADMETOX analysis

**Table 4:** ADME/ADMETox Analysis of GA (Generated using Schrödinger Maestro 2023/4 Licensed Version)

Molecule	The general name or identity of the molecule	GA
CNS	Central nervous system activity	−2
mol MW	Molecular weight	170.121
accptHB	The number of groups in the molecule capable of accepting hydrogen bonds	4.250
QPlogPC16	A prediction of how a molecule will partition between octanol and water, indicating its lipophilicity	6.464
QPlogPw	An estimation of the water partition coefficient	12.016
QPlogPo/w	A unified prediction considering both octanol and water partition coefficients	−0.585
QPlogS	A forecast of a compound's water solubility	−0.681
QPlogHERG	A forecast of the half-maximal inhibitory concentration for blocking the hERG K + channel	−1.396
QPPCaco	A forecast of a compound's permeability through Caco-2 cells, serving as a model for intestinal absorption	62.749
QPlogBB	A forecast of a compound's capability to penetrate the blood–brain barrier	−1.659
QPlogKp	A forecast of a compound's permeability through the skin	−5.488
QPlogKhsa	A forecast of a compound's binding affinity to human serum albumin	−0.987
RuleOfFive	A set of guidelines to evaluate drug-likeness, providing an indication of whether a compound is likely to possess favorable pharmacokinetic properties	0
RuleOfThree	A set of guidelines to evaluate lead-likeness, resembling RuleOfFive but tailored for early stages of drug discovery	1
Percent Human Oral Absorption	The percentage of oral absorption in humans	41.441

### Molecular dynamics analysis of GA with KEAP1: Insights from simulation details and results

In exploring the interaction dynamics between GA and KEAP1 through molecular dynamics simulation, the following findings were observed:

#### The Root Mean Square Deviation (RMSD) graph

**Fig. 9:**
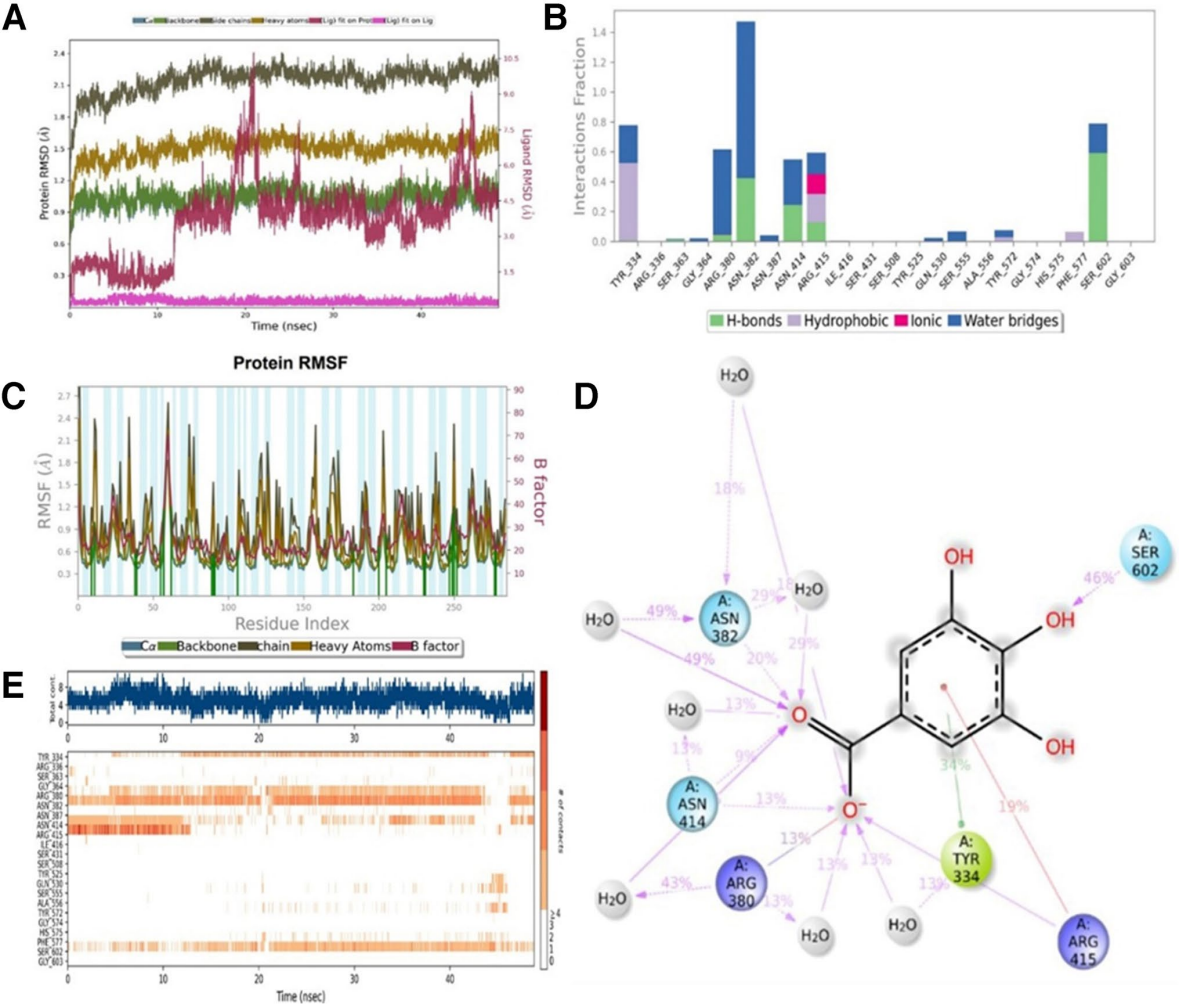
**A** The Root Mean Square Deviation (RMSD): After 12 ns, RMSD indicates the ligand has bound to the protein backbone. **B** Protein–ligand contacts (including hydrogen bonds, hydrophobic interactions, ionic bonds, and water bridges). **C** Ligand Root Mean Square Fluctuation (L-RMSF) characterizes fluctuations in ligand atom positions. **D** Schematic detailing ligand atom interactions with protein residues, showing interactions occurring more than 8.0% of the simulation time in the trajectory (0.00 to 48.80 nsec). (Images generated with Schrödinger Maestro 2023/1 Licensed version). **E** Timeline representation summarizing interactions and contacts (H-bonds, hydrophobic, ionic, water bridges) described previously

After 15 ns, the ligand–protein interactions were strengthened, indicating a stable binding event. This highlights the dynamic effects of GA on KEAP1 (Fig. 9A).

#### Protein–ligand contacts

A complex network of interactions was observed, including hydrogen bonds, hydrophobic interactions, ionic bonds, and water bridges. This detailed information is crucial for understanding the molecular recognition process, revealing the specific atoms involved in the binding modes (Fig. 9B).

#### The Root Mean Square Fluctuation (RMSF) graph

The interactions between the ligand and the protein backbone varied throughout the simulation, revealing different contact points. This dynamic behavior illustrates the flexibility of the GA binding site (Fig. 9C).

#### Schematic of ligand atom interactions with protein residues

Visualizing detailed interactions between ligand atoms and specific protein residues facilitates understanding of the binding mechanism at the molecular level. This visual representation enhances comprehension of the crucial interactions influencing the stability of the GA-KEAP1 complex (Fig. 9D).

Detailed molecular dynamics analyses reveal the dynamic nature of GA's interaction with KEAP1. Observations supported by RMSD, protein–ligand contacts, RMSF, and detailed diagrams provide a comprehensive understanding of the interactions between GA and KEAP1. These findings are crucial for elucidating the regulatory mechanisms and therapeutic potential of the KEAP1/NRF2/ARE pathway.

## Discussion

ACR, a recognized carcinogen, poses significant health risks, particularly to lung tissue. Exposure induces oxidative stress and inflammation, manifesting in respiratory symptoms and heightened lung cancer risk (Bin-Jumah et al. 2021; Sengul et al. 2023). ACR enhances ROS production, promoting lipid peroxidation and leading to cellular damage marked by elevated MDA levels (Foroutanfar et al. 2020). Furthermore, ACR diminishes cellular antioxidants such as GSH, compromising defense mechanisms against oxidative stress (Sansano et al. 2017; Tong et al. 2004). The fluid lining the pulmonary alveolar wall contains high concentrations of enzymatic antioxidants and abundant GSH. Alveolar cells possess some of the highest inherent antioxidant activity in the body. Despite this, the delicate balance between oxidation and reduction in the lungs can be disrupted by factors such as inhaled oxidants, circulating oxidants in the bloodstream, and locally generated free radicals (Erdemli et al. 2022; Yedier et al. 2022; Hajimohammadi et al. 2020). GSH alone may be insufficient to cope with oxidative stress. Therefore, other crucial antioxidant enzymes within cells, such as SOD, GPx, and CAT, support GSH’s function. These enzymes assist in combating free radicals, neutralizing peroxides, and detoxifying harmful compounds. ACR exposure not only decreases GSH levels but may also affect these enzymes, weakening the mechanisms that regulate oxidative stress. Our findings demonstrate GA's efficacy in protecting against ACR-induced pulmonary damage by inhibiting MDA elevation and preserving GSH levels in lung tissue. Moreover, GA effectively restored antioxidant enzyme activities (SOD, GPx, CAT) compromised by ACR exposure, highlighting its role in maintaining redox homeostasis (Yesildag et al. 2022; Gelen et al. 2022). In toxicity studies induced by ACR in lung tissues, it has been reported that oxidative stress occurs due to an increase in ROS. It has been revealed that this leads to an increase in MDA levels and a decrease in GSH, SOD, GPx, and CAT levels (Foroutanfar et al. 2020; Sansano et al. 2017). In some experimental studies, the positive effects of GA, a strong polyphenol, on these oxidant and antioxidant parameters have been demonstrated thanks to its antioxidant activity. (Er et al. 2020; Badhani et al. 2015; Nikbakht et al. 2015; Kalantar et al. 2018). Our study investigated GA's potential against ACR-induced lung injury, revealing its ability to mitigate oxidative stress at the cellular level.

Furthermore, the NRF2/HO-1/NF-κB pathway is a prominent signaling pathway due to its vital role in cellular antioxidant response, anti-inflammatory response, mitigation of mitochondrial damage, regulation of calcium entry, and control of cell death. Nrf2 functions as a pivotal transcription factor crucial for counteracting oxidative stress by promoting the transcription of antioxidant proteins, particularly the heme oxygenase-1 (HO-1) enzyme. Under normal physiological conditions, Nrf2 remains bound to Keap-1 in the cytoplasm, thereby maintaining its inactivity. However, during oxidative stress conditions, the dissociation of Keap-1 from Nrf2 occurs, allowing Nrf2 to translocate into the nucleus (Sohrabi et al. 2021; Jia et al. 2020). In some experimental toxicity studies conducted with ACR, it has been shown that ACR causes decreases in Nrf2/HO-1 levels and triggers oxidative stress in tissues accordingly (Yesildag et al. 2022; Jia et al. 2020; Fratta Pasini et al. 2020). In addition, it has been determined that GA prevents oxidative damage caused by ROS in tissues by upregulating this pathway thanks to its strong antioxidant activity (Zhou et al. 2022). In this presented study, in parallel with the literature information, it has been determined that GA suppresses oxidative stress in lung tissues by upregulating the Nrf2/HO-1 signaling pathway decreased by ACR.

Moreover, the activation of Nrf2 exerts significant anti-inflammatory effects through its interaction with NF-κB. In conditions of oxidative stress, Nrf2 deficiency leads to increased cytokine production and elevated NF-κB activity. One of the major target genes of Nrf2, HO-1, plays a crucial role in inhibiting NF-κB signaling and protecting the lungs from the detrimental effects of reactive oxygen species (ROS) generation, inflammation, and oxidative stress. This intricate interplay between Nrf2 and NF-κB signaling is further influenced by the MAPK pathways. NF-κB and MAPK signaling pathways are crucial downstream regulators of inflammation and are implicated in various degenerative diseases. NF-κB is a key modulator of inflammatory responses, while TNF-α exacerbates inflammation by upregulating the production of pro-inflammatory cytokines such as IL-1β and IL-6. Oxidative stress, a primary trigger of inflammation, activates NF-κB (Ileriturk, et al. 2022). In this context, ACR activates NF-κB, production of pro-inflammatory cytokines, which subsequently triggers inflammation (Sengul, et al. 2021). Similarly, our findings demonstrated elevated levels of p38α MAPK and NF-κB in response to ACR exposure. Additionally, we observed heightened levels of TNF-α, IL-1β, and IL-6, along with increased COX-2 activity. Previous research has demonstrated that GA alleviates inflammation by inhibiting NF-κB and p38α MAPK activation (Chu et al. 2024). In our study, we observed that GA effectively protected against ACR-induced lung damage by reducing levels of p38α MAPK, JNK, and NF-κB. This finding highlights the potent anti-inflammatory effects of GA and underscores its ability to enhance the cellular defense system.

A key factor triggering this process is the activation of caspase-3. Previous studies have documented that ACR increases Bax levels and activates caspase-3, thereby initiating the apoptotic pathway by enhancing cytochrome c release and p38α MAPK activation, while also modulating Bcl-2 expression (Cicek, et al. 2022). Our findings align with these studies, demonstrating that GA effectively mitigates these effects by reducing Bax levels, inhibiting caspase-3 activation, and modulating Bcl-2 expression, thereby protecting against ACR-induced apoptosis and oxidative damage (Gur, et al. 2021). Our findings align with these studies, demonstrating that ACR induces the activation of Bax and caspase-3. Specifically, the activation of caspase-3, coupled with increased p38α MAPK activity, highlights their interconnected role. In contrast, GA effectively attenuated these effects by decreasing Bax levels, inhibiting caspase-3 activation, and regulating Bcl-2 expression.

Moreover, comprehensive in silico analysis underscores the dynamic nature of GA binding to KEAP1. The observed interactions, as evidenced by RMSD, protein–ligand contacts, RMSF, and detailed diagrams, contribute to a nuanced understanding of the molecular events supporting GA's interaction with KEAP1. This information is crucial for elucidating potential regulatory mechanisms and therapeutic implications within the context of the KEAP1/NRF2/ARE pathway (Hasanat et al. 2017; Chaudhari and Bari 2016; Fatima et al. 2023; Kumar et al. 2019; Cai et al. 2021).

In this presented study, molecular docking studies were performed to determine the protective effect of GA against the toxic effects of ACR on Nrf-2/HO-1/NFκB in lung tissue. It was determined that the negative binding energies originating from ARG 415, SER 363, ASN 414, and SER 602 from the complex formations of ACR/Nrf-2-HO-1-NFκB were quite low compared to the binding energy of GA. In molecular binding studies conducted with ACR-induced toxicity, it was also determined that the binding energies of CAT, SOD, and Nrf2-Keap-1 were quite low (Cengiz et al. 2022). This situation also reveals that ACR triggered oxidative stress and caused oxidative damage in the tissue in molecular binding studies.

## Conclusion

In conclusion, it was determined in this study that ACR may cause toxicity in lung tissues even when administered via IG route and therefore ACR taken via ingestion may cause toxicity in lung tissue by causing oxidative stress and inflammation. On the other hand, it was also determined that GA, known for its antioxidant and anti-inflammatory properties, provides effective protection against oxidative stress and inflammation in lung tissue caused by ACR. Computational analysis revealed that GA is an important component that regulates the KEAP1/NRF2/ARE pathway by triggering its dynamic binding to KEAP1. ADME/ADMETox analysis of GA conducted using Schrödinger Maestro shows that GA exhibits moderate intestinal permeability (QPPCaco: 62.749) and human oral absorption (41.441%), but has limited ability to cross the blood–brain barrier (QPlogBB: −1.659). Additionally, GA complies with Lipinski's Rule of Five (score: 0), indicating favorable drug like properties, but its predicted hERG inhibition potential (−1.396) requires further safety evaluation. These findings suggest that GA has therapeutic potential in preserving lung health, particularly in preventing lung injury due to heavy metal toxins commonly found in nature.

## Data Availability

All source data for this work (or generated in this study) are available upon reasonable request.
